# A mysterious risk factor for bone cement leakage into the spinal canal through the Batson vein during percutaneous kyphoplasty: a case control study

**DOI:** 10.1186/s12891-019-2807-6

**Published:** 2019-09-12

**Authors:** Shuai Zhang, Gao Ju Wang, Qing Wang, Jin Yang, Shuang Xu, Chao Hua Yang

**Affiliations:** Department of Spine Surgery, The Affiliated Hospital of Southwest Medical University, No. 25 Taiping St, Luzhou, 646000 Sichuan China

**Keywords:** Osteoporotic vertebral compression fracture, Posterior vertebral wall morphology, Percutaneous kyphoplasty, Bone cement leakage, Batson vein, Three-dimensional CT

## Abstract

**Background:**

Percutaneous kyphoplasty (PKP) can effectively treat osteoporotic vertebral compression fractures (OVCFs). Although satisfactory clinical outcomes can be achieved, bone cement leakage remains a primary complication of PKP. Previous studies have found many high risk factors for bone cement leakage into the spinal canal; however, less attention to the posterior wall morphologies of different vertebral bodies may be one reason for the leakage. Here, we investigated the effect of posterior vertebral wall morphology in OVCF patients on bone cement leakage into the spinal canal during PKP.

**Methods:**

Ninety-eight OVCF patients with plain computed tomography (CT) scans and three-dimensional (3D) reconstruction images from T6 to L5 were enrolled. 3D-CT and multiplanar reconstructions (MPR) were used to measure the concave posterior vertebral wall depth (PVWCD) and the corresponding midsagittal diameter of the nonfractured vertebral body (VBSD), and the PVWCD/VBSD ratio was calculated. All subjects were divided into the thoracic or lumbar groups based on the location of the measured vertebrae to observe the value and differences in the PVWCD between both groups. The differences in PVWCD and PVWCD/VBSD between the thoracic and lumbar groups were compared. Three hundred fifty-seven patients (548 vertebrae) who underwent PKP within the same period were also divided into the thoracic and lumbar groups. The maximal sagittal diameter (BCSD), the area of the bone cement intrusion into the spinal canal (BCA), and the spinal canal encroachment rate (BCA/SCA × 100%) were measured to investigate the effect of the thoracic and lumbar posterior vertebral wall morphologies on bone cement leakage into the spinal canal through the Batson vein during PKP.

**Results:**

The PVWCDs gradually deepened from T6 to T12 (mean, 4.6 mm); however, the values gradually became shallower from L1 to L5 (mean, 0.6 mm). The PVWCD/VBSD ratio was approximately 16% from T6 to T12 and significantly less at 3% from L1 to L5 (*P* < 0.05). The rate of bone cement leakage into the spinal canal through the Batson vein was 10.1% in the thoracic group and 3.7% in the lumbar group during PKP. In the thoracic group, the BCSD was 3.1 ± 0.5 mm, the BCA was 30.2 ± 3.8 mm^2^, and the BCA/SCA ratio was 17.2 ± 2.0%. In the lumbar group, the BCSD was 1.4 ± 0.3 mm, the BCA was 14.8 ± 2.2 mm^2^, and the BCA/SCA ratio was 7.4 ± 1.0%. The BCSD, BCA and BCA/SCA ratio were significantly higher in the thoracic group than in the lumbar group (*P* < 0.05).

**Conclusions:**

The PVWCD in the middle and lower thoracic vertebrae can help reduce bone cement leakage into the spinal canal by enabling avoiding bone cement distribution over the posterior 1/6 of the vertebral body during PKP. The effect of the difference between the thoracic and lumbar posterior vertebral wall morphology on bone cement leakage into the spinal canal through the Batson vein in OVCF patients during PKP is one reason that the rate of bone cement leakage into the thoracic spinal canal is significantly higher than that into the lumbar spinal canal.

## Background

Percutaneous kyphoplasty (PKP) is an effective method for treating osteoporotic vertebral compression fractures (OVCFs) [1–5]. Although satisfactory clinical outcomes can be achieved, bone cement leakage remains a primary complication of PKP [6–8]. Bone cement can leak into the spinal canal, producing prolonged mechanical pressure. Bioheating and monomer polymerization of the bone cement will also release toxic substances, which can damage different levels of the spinal cord or nerve roots, potentially with disastrous consequences [9, 10]. Previous studies in the literature describe many high risk factors for bone cement leakage into the spinal canal, such as the type of vertebral fracture, the surgical procedure, the degree of preoperative vertebral body collapse, the bone cement injection volume, the opportunity for bone cement injection, and the relationship between the bone cement injection location and the corresponding vertebral venous system [7, 9–12]. We found that the posterior wall morphology was curved at the thoracic vertebra, which could hinder observation of bone cement leakage into the spinal canal. Therefore, we hypothesized that this curved posterior wall morphology might be a crucial risk factor for bone cement leakage into the spinal canal. Previous literature suggested that less attention to the posterior wall morphologies of different vertebral bodies may be one reason for bone cement leakage into the spinal canal.

Scholars have studied the vertebral and spinal canal morphologies by observing spinal specimens, plain X-ray films, plain computed tomography (CT) scans and three-dimensional (3D) reconstructions. To date, no relevant reports have been published that have quantitatively evaluated the differences in thoracic and lumbar posterior vertebral wall morphology [6, 13–18]. Therefore, in this study, we analyzed the reason that more bone cement leaks into the spinal canal in the thoracic region than in the lumbar region by measuring the concave posterior vertebral wall depth in the thoracic and lumbar regions via 3D-CT and multiplanar reconstruction (MPR).

## Methods

### Measurements and posterior wall morphology parameters of the thoracic and lumbar vertebral bodies

Patients who underwent PKP in our hospital from January 2008 to June 2017 and had both complete detailed clinical data and 3D-CT reconstructions were recruited for the study. All patients underwent a 64-slice plain CT scan from T6 to L5 (Light Speed VCT, GE Healthcare, Indiana, IN), with a 0.625-mm layer thickness. All original CT image data were transmitted to an ADM 4.4 Workstation for reconstruction and measurement (Advantage Workstation, version ADW 4.4, GE Healthcare, Indiana, IN). The inclusion criteria of the measured vertebral bodies were as follows: without acute or old fractures, without infectious or cancerous bone destruction, without hemivertebrae or congenital fusion vertebrae, and without metabolic osteopathy such as fibrous dysplasia or diffuse idiopathic skeletal hyperostosis. The exclusion criteria were vertebral bodies that had been treated with PKP.

**Fig. 1:**
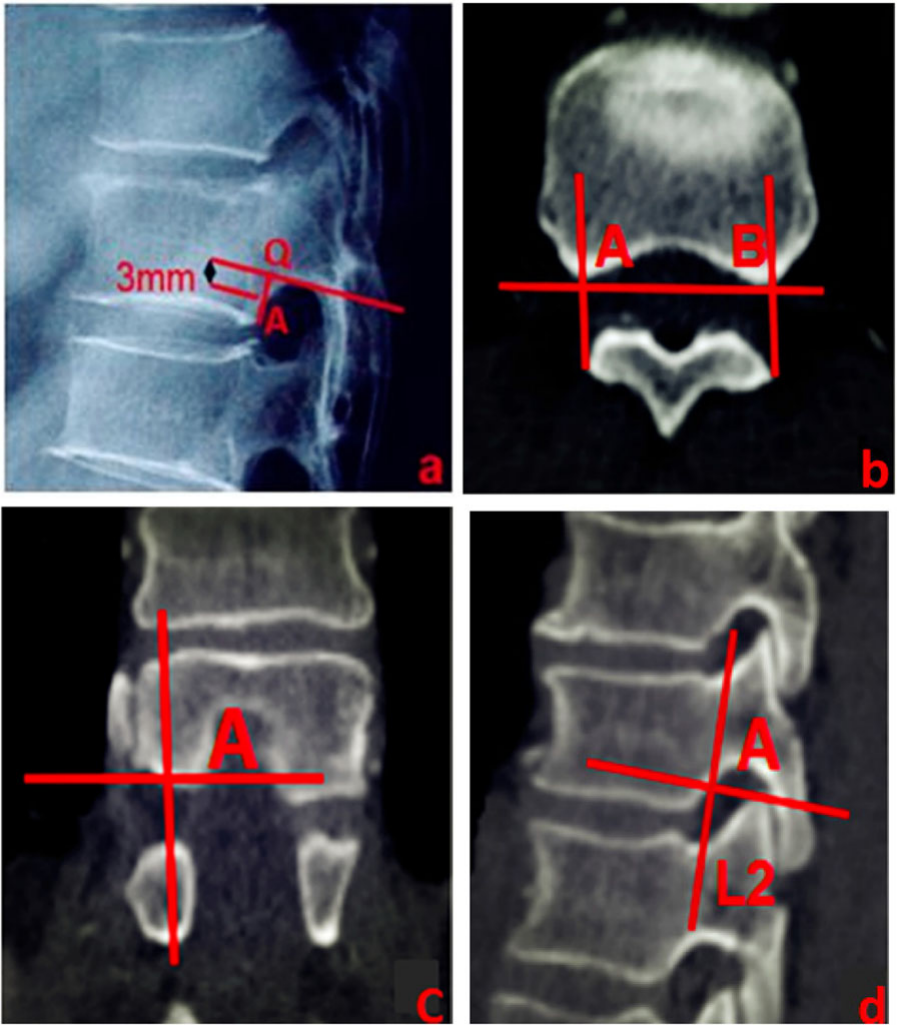
**a** Cross inferior vertebral notch to make the corresponding parallel line of the inferior endplate of the vertebral body and intersect the vertebral body at point Q. Point A, 3 mm below point Q, was selected as the observation point on the posterior vertebral body margin to determine whether bone cement entered the spinal canal. **b** Posterior margin bone cortex points A and B of the thoracic vertebral body were determined on the axial CT view at the same level as in a. **c** Point A was reconfirmed on the coronal view by a synchronous localization technique. **d** Line L2 at point A is perpendicular to the inferior endplate on the sagittal view

As in PKP, the C-arm X-ray machine was used to perform targeted fluoroscopy of fractured vertebral bodies on the CT positioning images, ensuring as much as possible that the upper and lower endplates of the targeted vertebral bodies were parallel to each other without disc signs and that the posterior edges of the vertebral bodies overlapped each other without bilateral signs. A cross inferior vertebral notch was used to make the corresponding parallel line of the inferior endplate of the vertebral body and intersect the vertebral body at point Q. To avoid the influence of the vertebral body and pedicle migration junction, point A, 3 mm below point Q, was selected as the observation point on the posterior vertebral body margin to determine whether bone cement entered the spinal canal. The cortical bone points A and B of the posterior border of the bilateral vertebral bodies were simultaneously determined on the axial CT image of this point. The synchronous positioning technology of the volume reconstruction interface of the ADM 4.4 Workstation was used to adjust the synchronous positioning coordinates on the coronal image to confirm the accuracy of the positions of points A and B and then connect A to B. The line, L2, was made perpendicular to the lower endplate on the sagittal image at point A (i.e., the posterior border of the vertebral body, which is usually used to determine bone cement leakage into the spinal canal in PKP; Fig. 1).

**Fig. 2:**
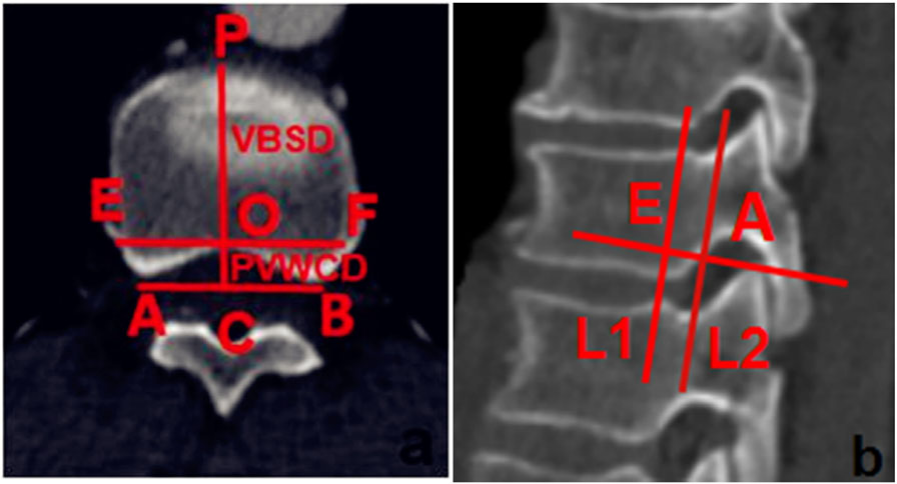
**a** Thoracic data measurement schematic diagram on the axial view showing lines PVWCD and VBSD; PVWCD represents the depth of the concave posterior vertebral wall; VBSD represents the mid-sagittal diameter of the nonfractured vertebral body. **b** Lines L1 and L2 are shown on the sagittal view; L1 corresponds to the true projection line of the posterior wall of the thoracic vertebral body, while L2 corresponds to the posterior border of the thoracic vertebrae on the lateral C-arm fluoroscopic view

On the axial CT image, apex O of the bony spinal canal protruding forward was determined, and a vertical line was made from O to AB, intersecting at point C. The distance between points A and C represented the concave posterior vertebral wall depth (PVWCD). The line EF parallel to AB and passing through O intersected with the lateral walls of both sides of the vertebral body at points E and F, respectively. On the lateral image, E was used to make line L1 parallel to L2 (the actual projection position of the line connecting the apexes of the posterior wall concave to the vertebral body on the lateral image; Fig. 2).

On the axial image, apex P of the leading-most edge of the vertebral body was determined, and the perpendicular line PC passing through point P was taken as AB. The distance between points P and C represented the mid-sagittal diameter of the vertebral body (VBSD; Fig. 2).

**Fig. 3:**
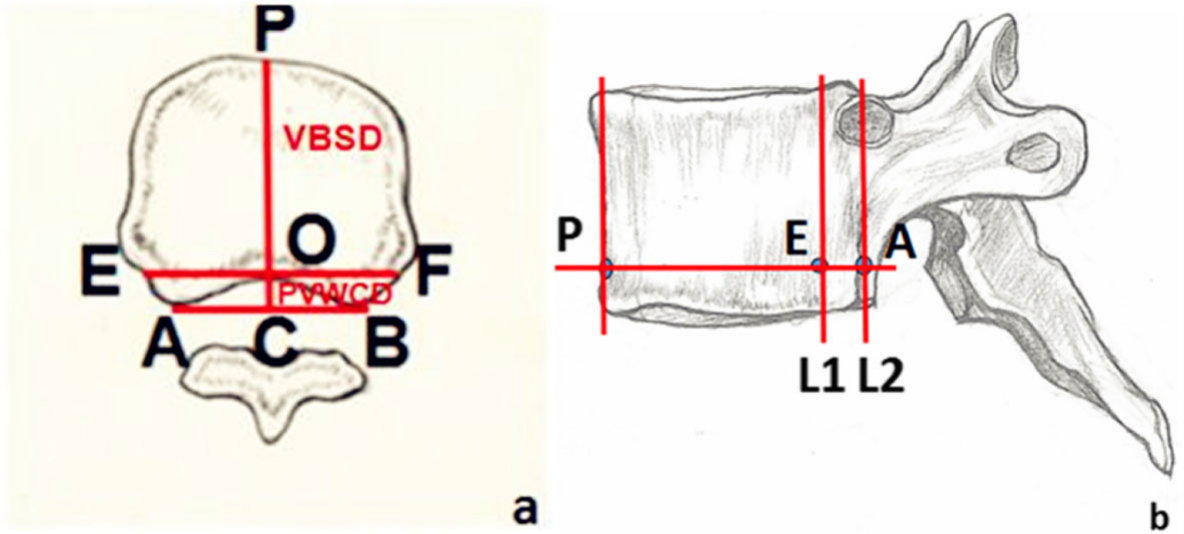
Data measurement schematic of the thoracic vertebrae. **a** Axial view of the thoracic data measurement schematic showing lines PVWCD and VBSD. PVWCD represents the depth of the concave posterior vertebral wall; VBSD represents the mid-sagittal diameter of the nonfractured vertebral body. **b** Sagittal view of the thoracic data measurement schematic shows lines L1 and L2. L1 corresponds to the true projection line of the posterior wall of the thoracic vertebra, while L2 corresponds to the posterior border of the vertebral body on the lateral C-arm X-ray view

The PVWCD, the mid-sagittal diameter of the same vertebral body (VBSD), and the PVWCD/VBSD ratio (PVWCD/VBSD× 100%) were measured (Fig. 3).

### Evaluation and measurement of the extent of bone cement leakage into the spinal canal

We selected patients who underwent PKP during the same time period. In this study, all operations were performed under real-time monitoring of a C-arm X-ray machine (Ziehm Solo, Ziehm imagine GMBH, Germany). Bone cement was injected through a unilateral puncture; thoracic vertebrae were punctured using the in-out-in method of Ryu et al. [19] and the lumbar vertebrae were punctured using the transverse-pedicle approach reported by Wang et al. [20]. The operation site was postoperatively reviewed with positive side X-rays and CT, and the original CT data were transmitted to an ADW 4.4 Workstation for reconstruction and measurement. The positive lateral plain X-ray films and axial, sagittal and coronal CT images of the operation site were observed. Vertebral bodies with bone cement leakage into the spinal canal through the Batson vein were selected and measured according to the characteristics reported by Yeom et al. [7].

**Fig. 4:**
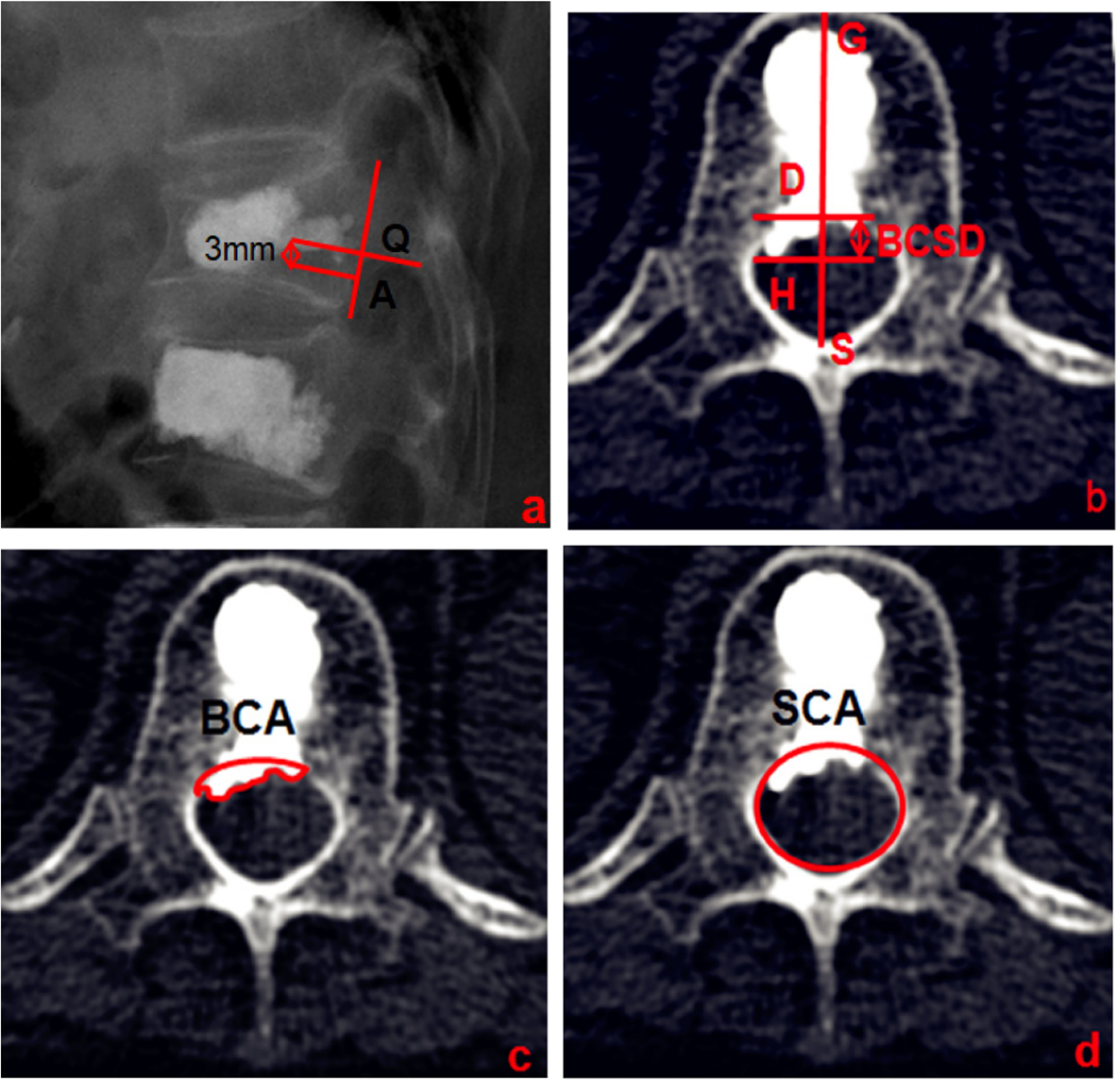
**a** Bone cement distribution near the L2 line. **b** CT transverse image of the vertebral body in figure a suggests bone cement leakage into the spinal canal. BCSD represents the maximum sagittal diameter of bone cement leakage into the spinal canal. **c** BCA shows the area of bone cement leakage into the spinal canal. **d** SCA shows the area of the bony spinal canal at the same level as b

The axial CT images were used to identify the plane of the largest area of bone cement invading the vertebral canal and determine the mid-sagittal diameter GS of the vertebral body (Fig. 4a). The GS intersected the anterior-most edge of the bony spinal canal at point D and crossed D to construct the perpendicular line, GS. The vertical line GS passing though the apex of the greatest extent of bone cement leakage into the spinal canal intersected at point H, and the distance of between points D and H was used to represent the maximal sagittal diameter of the bone cement leakage into the spinal canal (BCSD; Fig. 4b). The ADW 4.4 Workstation area measurement tool was used to measure the area of bone cement leakage into the spinal canal (BCA; Fig. 4c), the corresponding area of the bony spinal canal (SCA; Fig. 4d), and the spinal canal invasion rate (BCA /SCA × 100%) on the same levels.

### Statistical analysis

All data were statistically analyzed using SPSS 19.0 (Chicago, IL, USA). Data are presented as the means ± standard deviation for continuous variables and as the total number and proportion for categorical variables. Independent-sample t-tests were used to compare the differences in the PVWCD and PVWCD/VBSD between the thoracic and lumbar groups. The chi-square test was used to assess the difference in the incidence of bone cement leakage into the spinal canal between the thoracic and lumbar groups. Student’s t-test or the Wilcoxon rank-sum test were used to assess differences in the BCSD, BCA and BCA/SCA × 100% between the thoracic and lumbar groups. *P* < 0.05 was considered statistically significant.

## Results

### Measurements of parameters related to the vertebral body posterior wall morphology

Ninety-eight OVCF patients who underwent PKP at our hospital from January 2008 to June 2017 and had both complete detailed clinical data and 3D-CT reconstructions were recruited for the study. 31 men and 67 women, aged 58–89 years (average, 71.6 ± 1.2 years), were eligible for the study. The bone mineral density (BMD) of all subjects was − 3.2 ± 0.4 SD. A total of 1176 vertebral bodies were included in this study, but we only measured 1041 vertebral bodies (Table 1).

**Table 1:** Estimated and actual measurements

Vertebral body	Included amount(n)	Old fracture(n)	Fresh fracture (n)	Actual measurement(n)
T6	98	2	2	94
T7	98	5	3	90
T8	98	2	6	90
T9	98	1	3	94
T10	98	1	7	90
T11	98	2	6	90
T12	98	4	14	80
L1	98	6	22	70
L2	98	5	21	72
L3	98	2	7	89
L4	98	1	7	90
L5	98	0	6	92

**Fig. 5:**
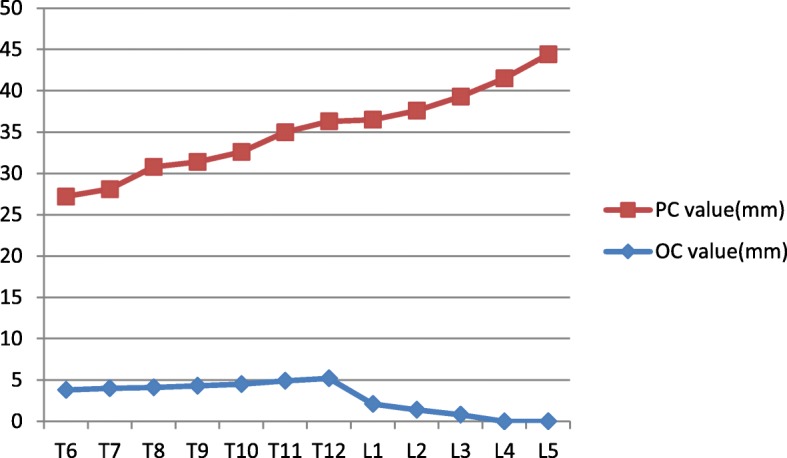
Trend chart of the PVWCD and VBSD values from T6 to L5. The PVWCD values increased from T6 to T12, significantly decreased from T12 to L1, and gradually decreased to almost 0 from L1 to L5. The VBSD values increased from T6 to L5

**Fig. 6:**
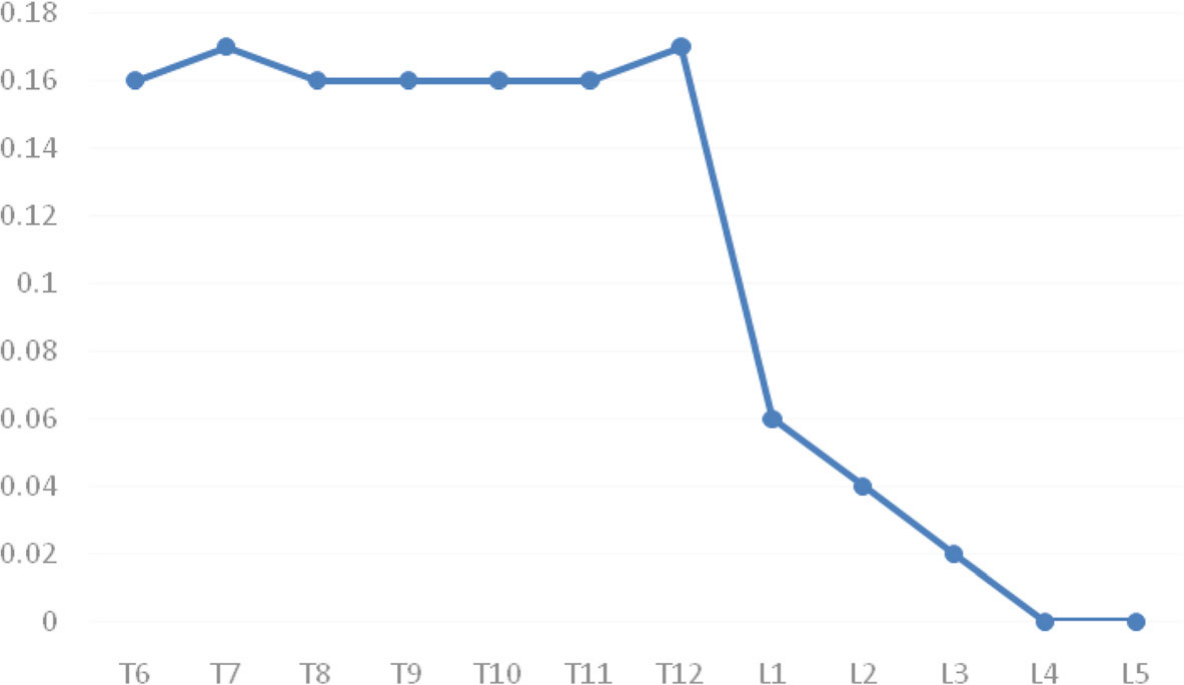
Trend chart of the PVWCD/VBSD ratios. The PVWCD/VBSD ratios were maintained at approximately 0.16 from T6 to T12, significantly reduced from T12 to L1, and gradually reduced to almost 0 from L1 to L5

Table 2 shows the PVWCD, VBSD, and PVWCD/VBSD× 100% parameters related to the posterior wall morphologies of the thoracic and lumbar vertebral bodies. The PVWCD gradually deepened from T6 to T12 (mean, 4.6 mm) but gradually became shallower from L1 to L5 (mean, 0.6 mm). The VBSD gradually increased from T6 to L5 (mean, 39.0 mm; Fig. 5). The PVWCD/VBSD ratio was approximately 16% from T6 to T12 but only 3% from L1 to L5 (Fig. 6). The PVWCD and PVWCD/VBSD in the lumbar group were significantly lower than those in the thoracic group (Table 2).

**Table 2:** Measurement data result

Vertebral body	PVWCD (mm)	VBSD (mm)	PVWCD/VBSD(100%)
Thoracic group			
T6(*n* = 94)	3.8 ± 0.1	23.4 ± 0.2	0.16 ± 0.01
T7(*n* = 90)	4.0 ± 0.1	24.1 ± 0.3	0.17 ± 0.02
T8(*n* = 90)	4.1 ± 0.1	26.7 ± 0.4	0.16 ± 0.00
T9(n = 94)	4.3 ± 0.1	27.1 ± 0.4	0.16 ± 0.01
T10(n = 90)	4.5 ± 0.1	28.1 ± 0.3	0.16 ± 0.02
T11(n = 90)	4.9 ± 0.1	30.1 ± 0.3	0.16 ± 0.03
T12(*n* = 80)	5.2 ± 0.1	31.1 ± 0.4	0.17 ± 0.01
Lumbar group			
L1(*n* = 70)	2.1 ± 0.1	34.4 ± 2.6	0.06 ± 0.00
L2(*n* = 72)	1.4 ± 0.0	36.2 ± 3.7	0.04 ± 0.00
L3(*n* = 89)	0.8 ± 0.0	38.5 ± 3.7	0.02 ± 0.00
L4(n = 90)	–	41.5 ± 3.2	–
L5(*n* = 92)	–	44.4 ± 4.5	–
T	71.800^*^		45.722^*^
P	0.000		0.000

Note: PVWCD represents the depth of the concave posterior vertebral wall. VBSD represents the Mid-sagittal diameter of the non-fracture vertebral body. Mark -- indicates that the value was almost 0 and could not be accurately measured by CT

^*^Compared with the thoracic vertebral group, *P* < 0.05

### Measurements of the parameters related to the degree of bone cement invasion of spinal canal

Three hundred fifty-seven OVCF patients (548 vertebral bodies) underwent PKP in the same period. The subjects (132 men and 225 women) were all Chinese and aged 55–94 years (average, 72.6 ± 4.4 years). The disease course of all subjects ranged from one day to ten months (average 33.8 ± 6.1 days). In all, 304 thoracic vertebrae and 244 lumbar vertebrae were treated with PKP, and Table 2 shows the surgical site distribution. The bone cement volume injected into a single vertebral body during PKP ranged from 2.5–6.0 ml (average, 4.2 ± 0.2 ml) in the thoracic group and from 3.5–7.5 ml (average, 5.1 ± 0.5 ml) in the lumbar group.

Of the 548 vertebral bodies treated with PKP, 304 were thoracic vertebrae, and 31 had bone cement leakage into the spinal canal, with a leakage rate of 10.1%. The remaining 244 vertebrae were lumbar vertebrae, and 9 had bone cement leakage into the spinal canal, with a leakage rate of 3.7% (Table 3). The BCSD, BCA and BCA/SCA were significantly higher in the thoracic group than in the lumbar group (*P* < 0.05; Table 4).

**Table 3:** The frequency of bone cement leakage into the spinal canal

	T6	T7	T8	T9	T10	T11	T12	L1	L2	L3	L4	L5	Total
Single vertebra	6	4	12	8	20	32	41	39	30	16	15	11	234
Double vertebrae	5	7	8	11	13	8	30	24	22	16	11	9	164
Three vertebrae	2	5	3	5	9	11	10	9	5	2	1	1	63
Four vertebrae	1	2	3	3	5	8	11	8	5	3	1	2	52
Five vertebrae	1	2	2	2	3	5	6	4	4	1	3	2	35
Total	15	20	28	29	50	64	98	84	66	38	31	25	548
The amounts of bone cement leakage(n)	1	2	2	3	5	7	11	5	2	1	1	0	40
The rates of bone cement leakage (%)	6.7	10.0	7.1	10.3	10.0	10.9	11.2	5.9	3.0	2.7	3.2	0	7.3

**Table 4:** The extent of bone cement intrusion into the spinal canal after PKP in two groups was compared

Groups	BCSD (mm)	BCA (mm^2^)	SCA (mm^2^)	(BCA /SCA) × 100%
T- group(*n* = 31)	3.1 ± 0.5	30.2 ± 3.8	175.7 ± 7.7	17.2 ± 2.0
L- group(n = 9)	1.4 ± 0.3	14.8 ± 2.2	199.4 ± 6.4	7.4 ± 1.0
T/Ζ	10.402	11.618	−8.394	−4.518
P	0.000*	0.000*	0.000*	0.000*

Note: BCSD represents the maximum depth of cement leakage into the spinal canal. BCA represents the area of bone cement intrusion into the spinal canal. SCA represents the the area of spinal canal at the same level

^*^Compared with the thoracic vertebral group, *P* < 0.05

## Discussion

PKP is one of the main treatments for OVCF, it can immediately reconstruct spinal stability and restore vertebral height, but bone cement leakage into the spinal canal is a complication that restricts the clinical application of PKP [6–8]. To reduce the incidence of cement leakage, many scholars have studied puncture techniques and the bone cement injection timing and amount [9–12]. Clinical observation revealed that the thoracic vertebral canal was oval; the posterior wall of the vertebral body was arched and concave into the vertebra; the boundary between the pedicle and vertebral body was unclear, and some ribs passed through the lateral portion of the pedicle. However, the lumbar vertebral canal was inverted in a triangle or clover shape; the posterior wall of the vertebral body was planar, and the boundary between the pedicle and vertebral body was clear. We hypothesized that the difference in the posterior wall morphology between the thoracic and lumbar vertebrae might be a crucial risk factor for bone cement leakage into the spinal canal. However, no relevant reports have been published that have quantitatively evaluated the differences in thoracic and lumbar posterior vertebral wall morphologies. In this study, we used CT and 3D reconstruction techniques to observe the morphological differences in the posterior wall between the thoracic and lumbar vertebrae, providing a new reference for preventing bone cement leakage into the spinal canal.

In this study, the average depth of the concave posterior vertebral wall was 4.6 mm, and the percentage of the depth of the concave posterior vertebral wall to the mid-sagittal diameter of the same vertebral body was approximately 16% (1/6) from T6 to T12. The average depth of the lumbar posterior wall concave into the vertebral body was only 0.6 mm. The percentage of the lumbar posterior wall concave into the vertebral body in the sagittal diameter of the same vertebral body was significantly less than 16% of the thoracic vertebral body. We found that because of the depression in the posterior wall of the vertebral bodies in the middle and lower thoracic vertebrae, the true posterior border of the vertebral body on the lateral image should be the projection of the anterior wall of the spinal canal on the surface of the vertebral body (L1), and the overlapping line of the posterior bone cortex of the bilateral vertebral bodies at the lower edge of the pedicle (L2) should not be considered a reference. Our results showed that during PKP, clinicians should avoid distributing the bone cement exceeding the posterior 1/6 of the vertebral body as much as possible to effectively prevent bone cement leakage into the spinal canal.

We retrospectively analyzed the imaging data for OVCF patients after PKP in our department. Under C-arm X-ray monitoring, line L2 was used to judge the occurrence of bone cement leakage into the spinal canal. We found that both the incidence and the extent of bone cement leakage invading the spinal canal in the thoracic group were significantly higher than those in the lumbar group (10.1% vs 3.7 and 22.5% vs 11.4, respectively). This may have been because the operator often used L2 under C-arm X-ray monitoring as a projection of the posterior wall of the vertebral body, ignoring the concave structure of the posterior wall of the middle and lower thoracic vertebrae. Thus, when the distribution of bone cement reached the posterior 1/6 of the middle and lower thoracic vertebrae, it leaked into the vertebral canal. Conversely, in the lumbar spine, because the structure was not concave, even if the bone cement distribution reached the posterior wall, it did not leak into the spinal canal. This observation further confirmed that the morphological characteristics of the posterior wall of the middle and lower thoracic vertebrae may be one risk factor for bone cement leakage into the spinal canal during PKP.

This study had some limitations. First, this study was a single-center imaging study; thus, it requires further validation with large multicenter samples. Second, this was a retrospective imaging study. The validity and reliability of defining the safe zone of bone cement injection guided by the morphological characteristics of the posterior wall of the middle and lower thoracic vertebrae should be further verified in clinical applications. Finally, the measured vertebral bodies selected in this study were all osteoporotic. The shape of these vertebral bodies and spinal canals differs from that of normal bone masses. Therefore, the data obtained in this study are only applicable to patients with OVCF.

## Data Availability

Data will be available upon request to the first author SZ.

## Abbreviations

3D: Three-dimensional
BCA: Area of bone cement intrusion into the spinal canal
BCSD: Maximal sagittal diameter of bone cement intrusion into the spinal canal
BMD: Bone mineral density
CT: Computed tomography scans
MPR: Multiplanar reconstruction
MRI: Magnetic resonance imaging
OVCF: Osteoporotic vertebral compression fracture
PKP: Percutaneous kyphoplasty
PVWCD: Concave posterior vertebral wall depth
SCA: Area of the spinal canal
VBSD: Mid-sagittal diameter of the nonfractured vertebral body
